# Terapia de Fotobiomodulação com Reabilitação Cardíaca em ICFEr/ICFEm: Promessa, Parâmetros e a Primazia do Exercício

**DOI:** 10.36660/abc.20250677

**Published:** 2025-12-11

**Authors:** Ricardo Carvalheiro, Ana Raquel Santos, António Valentim Gonçalves

**Affiliations:** 1 Unidade Local de Saúde de São José Hospital de Santa Marta Departamento de Cardiologia Lisboa Portugal Departamento de Cardiologia, Hospital de Santa Marta, Unidade Local de Saúde de São José, Centro Clínico Académico de Lisboa, Lisboa – Portugal

**Keywords:** Terapia de Fotobiomodulação, Teste Cardiopulmonar de Exercício, Reabilitação Cardíaca, Treinamento Intervalado de Alta Intensidade, Insuficiência Cardíaca com Fração de Ejeção Reduzida

A intolerância ao exercício na insuficiência cardíaca (IC) não é apenas um problema de bomba central. Mecanismos periféricos, como a extração prejudicada de oxigênio pelo músculo esquelético e a disfunção mitocondrial, são importantes fatores limitantes e se correlacionam com fadiga precoce e desfechos mais desfavoráveis.^1^ Com base nisso, a terapia de fotobiomodulação com luz/laser de baixa intensidade (PBMT) atraiu interesse como um complemento à reabilitação: ao atingir a citocromo-c oxidase, a PBMT pode aumentar a geração de ATP mitocondrial, modular a sinalização redox e melhorar a perfusão microvascular.^2^

Nesta edição, o artigo de Busin et al.^3^ avalia se a adição de PBMT ao treinamento contínuo de intensidade moderada (CMT) ou ao treinamento intervalado de alta intensidade (HIIT) aumenta os ganhos no consumo de oxigênio de pico (VO_2_pico) e outros índices de TECP entre pacientes com ICFEr/ICFEm sintomáticos e clinicamente estáveis. Em um delineamento não randomizado de cinco braços (CMT, HIIT, CMT+PBMT, HIIT+PBMT e um grupo controle) com 49 participantes, os autores observaram melhorias significativas na tolerância ao exercício em todos os grupos de intervenção, mas nenhum benefício adicional do PBMT no VO_2_pico, eficiência ventilatória (inclinação VE/VCO_2_) ou inclinação da eficiência do consumo de oxigênio (OUES). As tendências numéricas favoreceram alguns braços do PBMT, mas as diferenças entre os grupos não foram estatisticamente significativas. Os braços do HIIT tiveram um desempenho particularmente bom, mas o PBMT (aplicado apenas antes das sessões e em uma dose modesta) não produziu melhorias adicionais.

Como os médicos devem interpretar um sinal negativo de PBMT em conjunto com melhorias consistentes relacionadas ao treinamento? Primeiro, os efeitos do treinamento são a principal história – e uma história tranquilizadora. Décadas de trabalho mostram que o exercício estruturado na ICFEr melhora o VO_2_ pico, a capacidade funcional, a qualidade de vida e os marcadores prognósticos (incluindo OUES e VE/VCO_2_).^4,5^ Sínteses contemporâneas em fenótipos de IC reafirmam benefícios clinicamente significativos em desfechos funcionais.^6^ As recomendações das diretrizes permanecem claras: o treinamento físico é recomendado para melhorar o estado funcional e a qualidade de vida, e o encaminhamento para reabilitação cardíaca baseada em exercícios é apoiado em pacientes com IC elegíveis.^7,8^

Em segundo lugar, o contexto para o HIIT é misto. Dados randomizados e observacionais indicam que, em alguns programas, o HIIT pode gerar maiores melhorias no pico de VO_2_ do que o treinamento contínuo, enquanto outras experiências mostram diferenças mais modestas.^9^ Uma coorte de 10 anos de um único centro relatou associações entre exposição ao HIIT, melhora da remodelação ventricular esquerda e sobrevivência, mas o desenho não randomizado impede conclusões causais firmes.^10^ Sínteses mais amplas enfatizam que o exercício, em diferentes formatos, continua sendo central no tratamento da IC, e que o HIIT pode ser uma opção de alto rendimento quando individualizado, supervisionado e alinhado com considerações de segurança e adesão.^6^

Onde a PBMT se encaixa? Dados de atletas e voluntários saudáveis sugerem que a PBMT pode reduzir a fadiga e acelerar as adaptações ao treinamento, especialmente quando o tempo, a dose de energia e os pontos de irradiação são otimizados. Dados randomizados em participantes saudáveis relataram melhorias de resistência substancialmente mais rápidas quando a PBMT foi aplicada antes e depois das sessões de treinamento, e meta-análises, embora limitadas pela baixa qualidade geral dos estudos, sugerem benefícios para o desempenho e a fadiga.^11,12^ No entanto, a tradução para IC tem sido inconsistente. Em pacientes hospitalizados com ICFEr, a PBMT aguda reduziu o lactato e a percepção de esforço, sem melhorar a distância percorrida em seis minutos; em pacientes pós-cirurgia de revascularização do miocárdio, a PBMT não aumentou a capacidade funcional; e modelos animais de IC que associaram PBMT ao treinamento de resistência relataram melhores VO_2_ e tolerância.^13-15^

Os resultados atuais ampliam esse panorama misto: com PBMT pré-sessão (810 nm GaAlAs) em seis pontos bilaterais dos membros inferiores ao longo de dez semanas, não houve efeito aditivo no VO_2_pico, VE/VCO_2_ ou OUES. Explicações plausíveis incluem subdosagem, tempo subótimo (como a ausência de irradiação pós-exercício), locais de irradiação insuficientes e poder estatístico limitado em uma amostra pequena e não randomizada. Diferenças na idade ou no condicionamento físico basal (particularmente no braço HIIT+PBMT) também podem confundir os ganhos percentuais aparentes no VO_2_pico, dada a variabilidade na treinabilidade relacionada à idade.^2^

Para pacientes elegíveis com ICFEr/ICFEm, o treinamento supervisionado — CMT ou HIIT, dependendo dos objetivos, segurança e logística — continua sendo um pilar fundamental que melhora os parâmetros do CPET, os sintomas e a qualidade de vida relacionada à saúde; os programas devem ser dimensionados e acessíveis de forma equitativa em todos os sistemas de saúde.^6-8^ Em contraste, a PBMT não deve ser incorporada à reabilitação cardíaca de rotina neste momento. O presente estudo não encontrou benefício incremental com o protocolo testado, em linha com estudos cardiovasculares anteriores negativos ou ambíguos. Até que estudos randomizados multicêntricos com poder estatístico adequado definam a dose, o momento (pré- e/ou pós-sessão), o comprimento de onda e o número de locais e estendam os resultados do CPET aos desfechos clínicos, a PBMT deve permanecer em fase experimental.

Assim, os próximos estudos devem utilizar um protocolo PBMT padronizado (comprimento de onda fixo, energia por ponto, número de locais e tempo pré/pós-sessão), predefinir desfechos de CPET (pico de VO_2_, inclinação VE/VCO_2_, OUES) e enriquecer o recrutamento com pacientes com limitações metabólicas periféricas claras (baixa extração de O_2_ ou alta ineficiência ventilatória). Mais importante ainda, essas hipóteses precisam ser confirmadas em ECRs multicêntricos maiores e com poder estatístico adequado, que incluam desfechos clínicos e centrados no paciente.

Em conclusão, este estudo reconfirma a eficácia robusta do exercício estruturado na ICFEr/ICFEi, especialmente quando o HIIT é viável, em consonância com as diretrizes internacionais. Ao mesmo tempo, modera o entusiasmo pelo PBMT na reabilitação clínica como atualmente oferecida. A justificativa mitocondrial permanece convincente; a receita clínica, ainda não.
