# Supplementary material for: Terapia de Fotobiomodulação com Reabilitação Cardíaca em ICFEr/ICFEm: Promessa, Parâmetros e a Primazia do Exercício
Source: Arq Bras Cardiol. 2025 Dec 11;122(11):e20250677. [Article in Portuguese] doi: 10.36660/abc.20250677 (PMC12978275; doi:10.36660/abc.20250677)
Supplement: Translation

# Translation: Photobiomodulation Therapy with Cardiac Rehabilitation in HFrEF/HFmrEF: Promise, Parameters, and the Primacy of Exercise

**DOI:** 10.36660/abc.20250677i

**Authors:** Ricardo Carvalheiro, Ana Raquel Santos, António Valentim Gonçalves

**Affiliations:** 1 Lisboa Portugal Departamento de Cardiologia, Hospital de Santa Marta, Unidade Local de Saúde de São José, Centro Clínico Académico de Lisboa, Lisboa – Portugal

**Keywords:** Photobiomodulation Therapy, Cardiopulmonary Exercise Testing, Cardiac Rehabilitation, High-intensity Interval Training, Heart Failure with Reduced Ejection Fraction

Exercise intolerance in heart failure (HF) is not solely a central pump problem. Peripheral mechanisms, such as impaired skeletal-muscle oxygen extraction and mitochondrial dysfunction, are major limiting factors and correlate with early fatigue and poorer outcomes.^1^ On this basis, low-level light/laser photobiomodulation therapy (PBMT) has attracted interest as an adjunct to rehabilitation: by targeting cytochrome-c oxidase, PBMT may boost mitochondrial ATP generation, modulate redox signaling, and enhance microvascular perfusion.^2^

In this edition, the article by Busin et al.^3^ evaluates whether the addition of PBMT to either continuous moderate-intensity training (CMT) or high-intensity interval training (HIIT) augments gains in peak oxygen uptake (VO_2_peak) and other CPET indices among symptomatic, clinically stable HFrEF/HFmrEF patients. In a non-randomized, five-arm design (CMT, HIIT, CMT+PBMT, HIIT+PBMT, and a control group) with 49 participants, the authors observed significant improvements in exercise tolerance across all intervention groups, but no additional benefit from PBMT on VO_2_peak, ventilatory efficiency (VE/VCO_2_ slope), or oxygen uptake efficiency slope (OUES). Numerical trends favored some PBMT arms, yet between-group differences were not statistically significant. The HIIT arms performed particularly well, but PBMT (applied only before sessions and at a modest dose) did not yield additional improvements.

How should clinicians interpret a negative PBMT signal alongside consistent training-related improvements? First, the training effects are the main story - and a reassuring one. Decades of work show that structured exercise in HFrEF improves VO_2_ peak, functional capacity, quality of life, and prognostic markers (including OUES and VE/VCO_2_).^4,5^ Contemporary syntheses across HF phenotypes reaffirm clinically meaningful benefits on functional endpoints.^6^ Guideline recommendations remain clear: exercise training is recommended to improve functional status and quality of life, and referral to exercise-based cardiac rehabilitation is supported in eligible HF patients.^7,8^

Second, the context for HIIT is mixed. Randomized and observational data indicate that, in some programs, HIIT can yield greater VO_2_ peak improvements than continuous training, while other experiences show more modest differences.^9^ A 10-year single-center cohort reported associations between HIIT exposure, improved left-ventricular remodeling, and survival, but the nonrandomized design precludes firm causal conclusions.^10^ Broader syntheses emphasize that exercise, across different formats, remains central in HF care, and that HIIT may be a high-yield option when individualized, supervised, and aligned with safety and adherence considerations.^6^

Where does PBMT fit? Athletic and healthy volunteer data suggest PBMT can reduce fatigue and accelerate training adaptations, especially when timing, energy dose, and irradiation points are optimized. Randomized data in healthy participants reported substantially faster endurance improvements when PBMT was applied before and after training sessions, and meta-analyses, although limited by overall low study quality, suggest benefits for performance and fatigue.^11,12^ Yet translation to HF has been inconsistent. In hospitalized HFrEF, acute PBMT reduced lactate and perceived exertion without improving six-minute walk distance; in post-CABG patients, PBMT did not increase functional capacity; and animal HF models pairing PBMT with resistance training reported better VO_2_ and tolerance.^13-15^

The present results extend that mixed picture: with pre-session PBMT (810 nm GaAlAs) to six bilateral lower-limb points over ten weeks, no additive effect emerged on VO_2_peak, VE/VCO_2_, or OUES. Plausible explanations include under-dosing, suboptimal timing (such as the absence of post-exercise irradiation), insufficient irradiation sites, and limited statistical power in a small, non-randomized sample. Differences in age or baseline fitness (particularly within the HIIT+PBMT arm) can also confound apparent percentage gains in VO_2_peak, given age-related variability in trainability.^2^

For eligible HFrEF/HFmrEF patients, supervised training — CMT or HIIT, depending on goals, safety, and logistics — remains a cornerstone that improves CPET parameters, symptoms, and health-related quality of life; programs should be scaled and equitably accessible across health systems.^6-8^ By contrast, PBMT should not be incorporated into routine cardiac rehabilitation at this time. The present study found no incremental benefit with the tested protocol, aligning with prior negative or equivocal cardiovascular trials. Until adequately powered multicenter randomized studies define the dose, timing (pre- and/or post-session), wavelength, and number of sites, and extend outcomes from CPET to clinical endpoints, PBMT should remain investigational.

Accordingly, next studies should use a standardized PBMT protocol (fixed wavelength, energy per point, number of sites, and pre/post-session timing), predefine CPET endpoints (VO_2_peak, VE/VCO_2_ slope, OUES), and enrich enrollment with patients who have clear peripheral metabolic limitations (low O_2_ extraction or high ventilatory inefficiency). Most importantly, these hypotheses need confirmation in larger, adequately powered multicenter RCTs that include patient-centered and clinical endpoints.

In conclusion, this study reconfirms the robust efficacy of structured exercise in HFrEF/HFmrEF, especially when HIIT is feasible, in step with international guidelines. At the same time, it tempers enthusiasm for PBMT in clinical rehabilitation as currently delivered. The mitochondrial rationale remains compelling; the clinical recipe does not - yet.
